# Supplementary figures and images for: Short cracks in knee meniscus tissue cause strain concentrations, but do not reduce ultimate stress, in single-cycle uniaxial tension
Source: R Soc Open Sci. 2018 Nov 14;5(11):181166. doi: 10.1098/rsos.181166 (PMC6281910; doi:10.1098/rsos.181166)

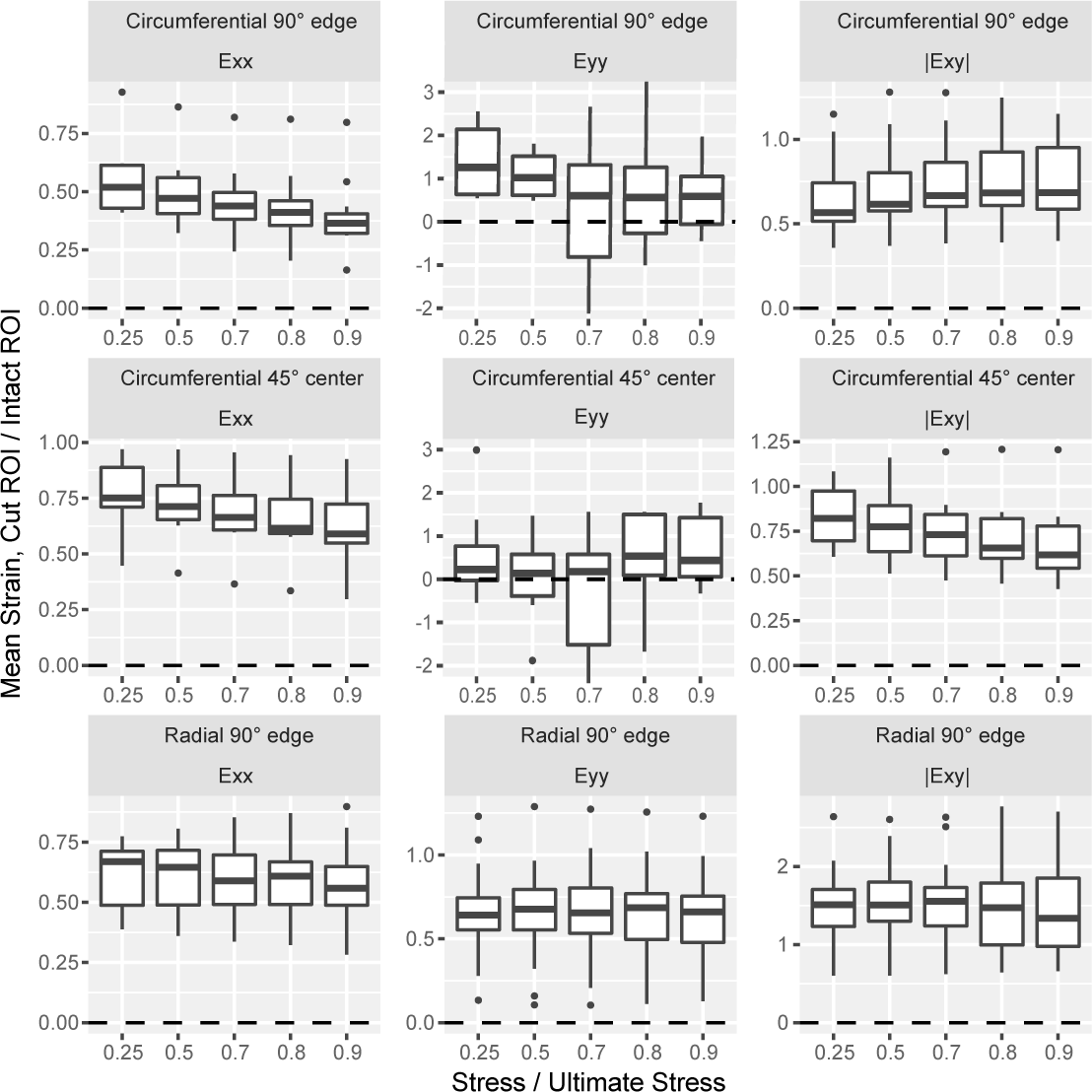

Supplement: Mean DIC strain; cut vs. intact ROI ratio [file rsos181166supp1.tif]
